# Engineering of Vaginal Lactobacilli to Express Fluorescent Proteins Enables the Analysis of Their Mixture in Nanofibers

**DOI:** 10.3390/ijms222413631

**Published:** 2021-12-20

**Authors:** Spase Stojanov, Tina Vida Plavec, Julijana Kristl, Špela Zupančič, Aleš Berlec

**Affiliations:** 1 Department of Biotechnology, Jožef Stefan Institute, SI-1000 Ljubljana, Slovenia; spase.stojanov@ijs.si (S.S.); tina.plavec@ijs.si (T.V.P.); 2 Faculty of Pharmacy, University of Ljubljana, SI-1000 Ljubljana, Slovenia; julijana.kristl@ffa.uni-lj.si (J.K.); spela.zupancic@ffa.uni-lj.si (Š.Z.)

**Keywords:** lactobacilli, vaginal probiotics, fluorescent proteins, electrospinning, nanofibers, probiotic analysis, probiotic delivery

## Abstract

Lactobacilli are a promising natural tool against vaginal dysbiosis and infections. However, new local delivery systems and additional knowledge about their distribution and mechanism of action would contribute to the development of effective medicine. This will be facilitated by the introduction of the techniques for effective, inexpensive, and real-time tracking of these probiotics following their release. Here, we engineered three model vaginal lactobacilli (*Lactobacillus crispatus* ATCC 33820, *Lactobacillus gasseri* ATCC 33323, and *Lactobacillus jensenii* ATCC 25258) and a control *Lactobacillus plantarum* ATCC 8014 to express fluorescent proteins with different spectral properties, including infrared fluorescent protein (IRFP), green fluorescent protein (GFP), red fluorescent protein (mCherry), and blue fluorescent protein (mTagBFP2). The expression of these fluorescent proteins differed between the *Lactobacillus* species and enabled quantification and discrimination between lactobacilli, with the longer wavelength fluorescent proteins showing superior resolving power. Each *Lactobacillus* strain was labeled with an individual fluorescent protein and incorporated into poly (ethylene oxide) nanofibers using electrospinning, as confirmed by fluorescence and scanning electron microscopy. The lactobacilli retained their fluorescence in nanofibers, as well as after nanofiber dissolution. To summarize, vaginal lactobacilli were incorporated into electrospun nanofibers to provide a potential solid vaginal delivery system, and the fluorescent proteins were introduced to distinguish between them and allow their tracking in the future probiotic-delivery studies.

## 1. Introduction

The healthy human vagina is home to around 50 microbial species, of which the most dominating are bacteria from the genus *Lactobacillus* [1]. The main ones found are *Lactobacillus crispatus*, *Lactobacillus jensenii*, *Lactobacillus gasseri*, and *Lactobacillus iners*. Each of these is dominant in its community type, and *L. crispatus* is the most abundant [2]. Vaginal infections are favored by dysbiosis of the normal vaginal microbiota, whereby the numbers of lactobacilli decrease. This can allow the overgrowth of several opportunistic pathogens, including *Gardnerella vaginalis*, *Atopobium vaginae*, and *Candida albicans* [3].

Antimicrobial drugs are the first line of defense against bacterial infections. However, their frequent use can lead to antimicrobial resistance and also to high infection recurrence rates (i.e., ~50%) [3]. These high recurrence rates appear to be associated with the nonselective mechanisms of the antimicrobial drugs, where as well as the pathogens, the normal lactobacilli are also reduced [4,5]. Therefore, new therapeutic strategies are required to normalize vaginal dysbiosis. Re-establishing the vaginal microbial balance with *Lactobacillus* bacteria as probiotics can then prevent the overgrowth of vaginal pathogens and thus prevent recurrence of the vaginal infection. The dominant vaginal species, *L. crispatus*, has been shown to be active against vaginal pathogens, both alone and in combination with other lactobacilli [6,7,8,9,10,11]. Antimicrobial properties of other vaginal lactobacilli against vaginal and uropathogens have also been reported [12,13,14,15].

Despite the beneficial properties of vaginal lactobacilli, their application as probiotics is limited by the lack of an appropriate delivery system and their low viability and high sensitivity to environmental factors [16]. The currently used liquid, semi-solid, and solid dosage forms for vaginal drug delivery have several limitations, including short residence time, discomfort, leakage, imprecise dosing, and variable drug distribution [17]. On the other hand, nanofibers have a high surface-to-volume ratio and can provide high drug loading, controlled release, cell binding, good bioavailability, and cost-effectiveness [18].

Nanofibers are produced by electrospinning, which is based on the drying of a thin liquid jet that is formed from a drop of polymer solution in a strong electric field [19] and are used in numerous applications [20,21]. They also represent an effective material for delivering different types of drugs to the nasal, oral, and vaginal mucosa [22], while at the same time, they protect drugs from environmental factors [23]. Different compounds can be incorporated into nanofibers, including small drug molecules, proteins, nucleic acids, and cells. *Lactobacillus* bacteria have also been incorporated into electrospun nanofibers, with *L. plantarum* being the most frequently used [24,25,26,27,28]. Other *Lactobacillus* species have also been electrospun using different polymers, namely, agrowaste amended with poly (vinyl alcohol) for *Lactobacillus acidophilus* [29], poly (vinyl alcohol) and sodium alginate for *Lactobacillus rhamnosus* [30], poly (vinyl alcohol) for *Lactobacillus gasseri* [31], and Eudragit L100 and sodium alginate for *Lactobacillus paracasei* [32]. In a recent study, we successfully incorporated nine different *Lactobacillus* species into polyethylene oxide (PEO) nanofibers (i.e., *L. acidophilus*, *L. delbrueckii* ssp. *bulgaricus*, *L. casei*, *L. gasseri*, *L. paracasei*, *L. plantarum*, *L. reuteri*, *L. rhamnosus*, *L. salivarius*) with high viability after electrospinning process [33]. However, few studies have reported incorporation of vaginal lactobacilli. One example was the incorporation of *L. gasseri* CRL1320 and *L. rhamnosus* CRL1332 into polyvinyl-alcohol nanofibers, with skimmed milk lactose and glycerol serving as bioprotective agents [34]. In another study, vaginal *L. acidophilus* was incorporated into polyvinyl alcohol and polyvinylpyrrolidone nanofibers [35].

Apart from the lack of delivery systems, vaginal probiotics use is hampered by the lack of research into their distribution and mechanism of action [36]. The spatial identification of individual strains in mixtures is particularly challenging, as stains (such as Syto 9) cannot be used to distinguish them, and custom antibodies against specific surface antigens or fluorescent in-situ hybridization have to be used instead [37]. Genetic engineering of lactobacilli for the production of fluorescent proteins is a rapid and effective method for tracking and distinguishing lactobacilli. Fluorescent proteins with different spectral properties have already been used to study the distribution and properties of lactobacilli [38,39,40,41,42]. However, few studies have reported the incorporation of fluorescent lactobacilli into nanofibers. In our recent study, *L. plantarum* expressing red fluorescent protein mCherry was incorporated into PEO nanofibers to evaluate nanofiber dissolution and lactobacilli release [27].

The aim and novelty of the present study was to engineer vaginal lactobacilli to express fluorescent proteins and incorporate them into nanofibers. Four different species of lactobacilli (*L. crispatus*, *L. gasseri*, *L. jensenii*, and *L. plantarum*) were genetically modified to express compatible fluorescent proteins with different spectral properties: infrared fluorescent protein (IRFP); green fluorescent protein (GFP); red fluorescent protein (mCherry); and blue fluorescent protein (mTagBFP2). Quantification of their fluorescence, the overlap between the fluorescences of the different fluorescent proteins, and the differentiation between the fluorescent species were evaluated. The four engineered species were mixed with PEO solution and electrospun into nanofibers as the potential delivery system. By using genetic engineering, we have introduced a new technique for effective, inexpensive, and real-time tracking of probiotics following their incorporation into nanofibers and nanofiber dissolution.

## 2. Results

### 2.1. Genetic Constructs for Expression of Fluorescent Proteins in Vaginal Lactobacilli

**Figure 1:**
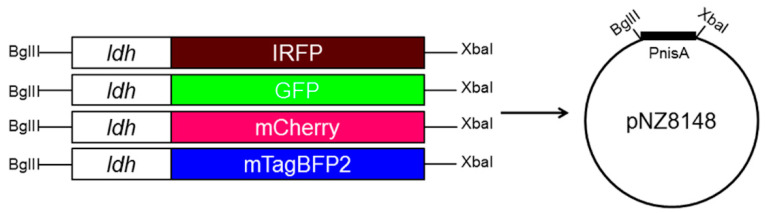
Assemblies of the *ldh* promoter and the four genes that encode the fluorescent proteins (IRFP, GFP, mCherry, mTagBFP2) in pNZ8148 plasmid. BglII, XbaI—restriction recognition sites that were used for cloning; PnisA—nisin promoter.

To complement the existing pNZ-ldh-GFP plasmid that encodes GFP under the control of the *ldh* promoter from *L. plantarum*, genes that encode the fluorescent proteins IRFP, mCherry, and mTagBFP2 were scarlessly fused with the *ldh* promoter using overlap-extension PCR (Figure 1). The gene fusions were cloned into the pNZ1848 plasmid, thereby replacing the inducible nisin promoter and yielding plasmids pNZ-ldh-IRFP, pNZ-ldh-mCherry, and pNZ-ldh-mTagBFP2. All four plasmids were separately transformed in all four *Lactobacillus* species, yielding 16 combinations (Table S1) and thus providing a wide range of possibilities to distinguish between them when used simultaneously. They were characterized in the following experiment with respect to the intensity of the expressed fluorescent proteins.

### 2.2. Analysis of Fluorescent Protein Expression in Vaginal Lactobacilli

**Figure 2:**
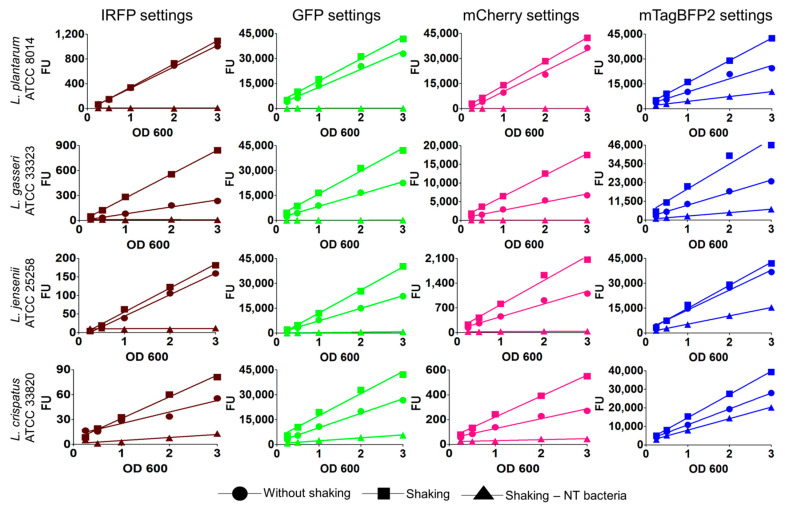
Concentration-dependent fluorescence of lactobacilli expressing different fluorescent proteins. Linear regression analyses of fluorescence and OD_600_ of lactobacilli grown at 37 °C without and with shaking (aeration) are shown. Nontransformed (NT) bacteria were used as controls. FU—fluorescence unit.

Fluorescent protein expression in lactobacilli was confirmed by measuring the fluorescence of all 16 bacterial suspensions and was shown to be appropriate for the detection of bacteria (Figure 2). The expression of the individual fluorescent proteins depended on the culture conditions and differed between the species. Growing the bacteria with shaking (aeration) resulted in higher fluorescence per unit OD_600_ in comparison to the samples grown without shaking (Figure 2) and significantly steeper slopes of regression lines (Table S1). This is in accordance with previous observations of oxygen-mediated post-translational activation of fluorescent proteins [43]. Fluorescent proteins GFP and TagBFP2 were expressed in all four strains at aerobic conditions, with 42,000 ± 2000 fluorescence units (FU) in suspensions at OD_600_ 3.0. By contrast, the expression of IRFP and mCherry was strain-dependent and was highest in *L. plantarum* (1100 FU for IRFP and 42,500 FU for mCherry). In comparison, the fluorescence of mCherry in other lactobacilli was lower by a factor of 2.4 for *L. gasseri*, a factor of 20.5 for *L. jensenii*, and a factor of 424 for *L. crispatus*. The fluorescence of these bacteria expressing the fluorescent proteins correlated linearly with a bacterial concentration in the OD_600_ range between 0.25 and 3.00 (Figure 2). The coefficients of determination (R^2^) for the engineered bacteria were above 0.96 with the exception of *L. plantarum* expressing mTagBFP2 and *L. crispatus* expressing IRFP, where R^2^ was lower (Supplementary Material: Table S1).

The nontransformed lactobacilli were used as controls because of the possibility of their autofluorescence. No or little concentration-dependent autofluorescence was seen for IRFP, GFP, and mCherry. Although bacteria showed relatively strong and concentration-dependent autofluorescence when measured using settings corresponding to mTagBFP2, the absolute fluorescence of nontransformed bacteria was significantly lower in comparison to the transformed bacteria.

**Figure 3:**
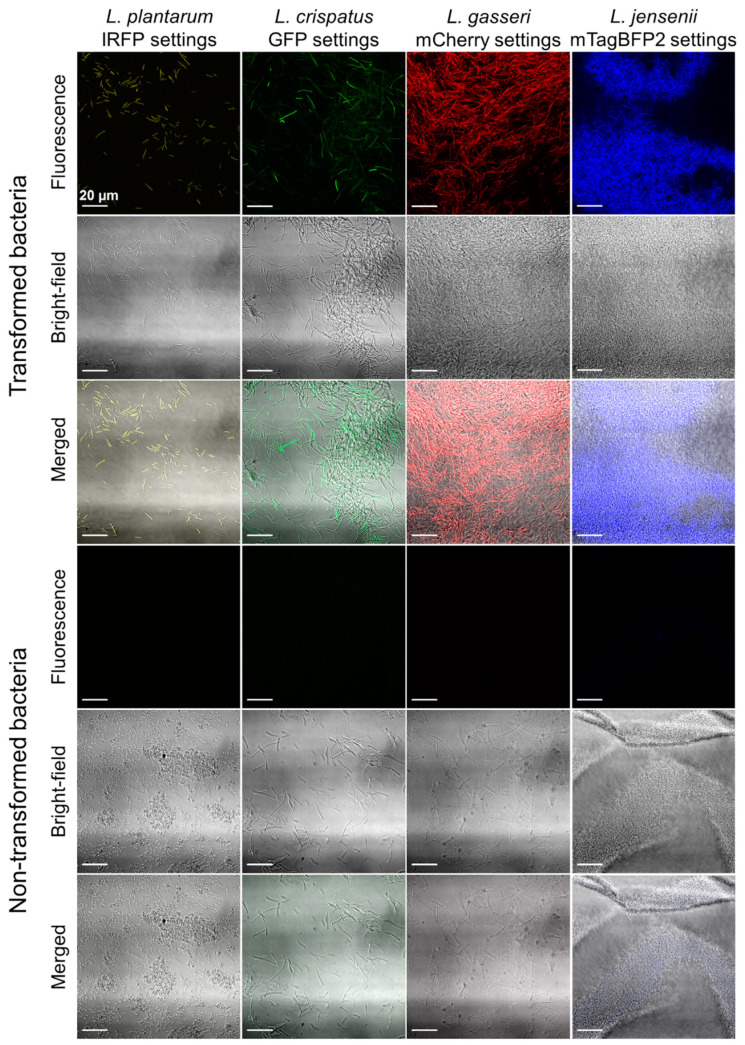
Representative confocal microscopy images of the lactobacilli expressing the different fluorescent proteins, as *L. plantarum* expressing IRFP, *L. gasseri* expressing mCherry, *L. crispatus* expressing GFP, and *L. jensenii* expressing mTagBFP2, in comparison to the nontransformed bacteria.

The expression of the fluorescent proteins in these vaginal lactobacilli was also detected under a confocal microscope (Figure 3). Fluorescence was detected for all the engineered lactobacilli, while no fluorescence was detected for the nontransformed lactobacilli when using the settings for IRFP and mCherry. Some autofluorescence was seen for the nontransformed bacteria when using the settings for GFP and mTagBFP2; however, the signals were lower in comparison to those of the engineered bacteria.

### 2.3. Fluorescence-Based Distinction between Lactobacilli in Mixture and Assessment of the Influence of Spectral Overlap

**Figure 4:**
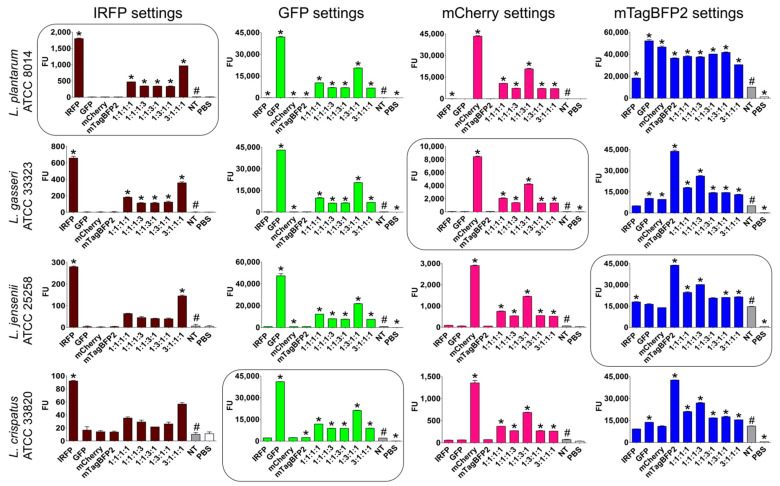
Distinction between the same species of *Lactobacillus* expressing different fluorescent proteins. Fluorescence was measured for the individual fluorescent strains and their mixtures, using settings corresponding to all four of the fluorescent proteins. The ratios indicate the proportions of the species expressing the fluorescent proteins in the following order: IRFP:GFP:mCherry:mTagBFP2. The encircled graphs represent the combination of fluorescent proteins and lactobacilli that were selected for further studies. * *p* < 0.05 (Student’s *t* tests) relative to nontransformed strain (NT, high-lighted with # for clarity). FU—fluorescence units; PBS—phosphate-buffered saline.

The individual *Lactobacillus* species were transformed with all four of the plasmids that encoded these fluorescent proteins with different spectral properties. Engineered strains of the same species of lactobacilli were mixed in different proportions, and their fluorescence was measured in a suspension (Figure 4). For the majority of the mixtures, the individual fluorescent strain was clearly distinguished by significantly higher fluorescence when using the settings corresponding to the fluorescent protein it expressed (i.e., the relevant excitation and emission wavelengths). Additionally, the fluorescence signals correlated with the content of the individual strain in the mixtures. The lowest specificity was observed with the mTagBFP2 settings, especially in the case of *L. plantarum*, where considerable fluorescence was also seen for other fluorescent proteins. This was attributed to shorter wavelengths and bacterial autofluorescence.

### 2.4. Selection and Analysis of Optimal Lactobacilli-Fluorescent Protein Combinations

**Figure 5:**
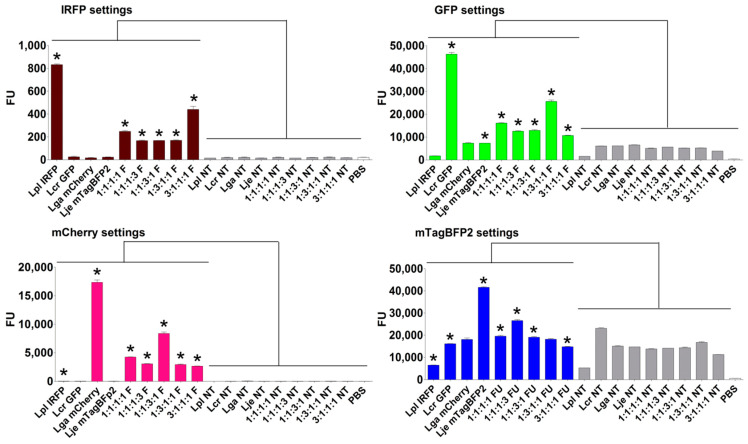
Fluorescence-based distinction of the different fluorescent lactobacilli and the nontransformed (NT) lactobacilli. Fluorescence was measured for the individual fluorescent species or their mixtures using the settings for all four of the fluorescent proteins. The ratios indicate the proportions of species in the mixtures in the following order: *L. plantarum* expressing IRFP; *L. crispatus* expressing GFP; *L. gasseri* expressing mCherry; and *L. jensenii* expressing mTagBFP2. * *p* < 0.05 (Student’s *t* tests), obtained by comparing fluorescent strain (F) to its nontransformed counterpart (NT). Lpl—*L. plantarum*; Lga—*L. gasseri*; Lcr—*L. crispatus*; Lje—*L. jensenii*; PBS—phosphate-buffered saline.

To enable the distinction between the *Lactobacillus* species in complex mixtures or probiotic products, and thereby facilitate characterization in further studies, each species was defined with an individual fluorescent protein on the basis of the fluorescence properties of all 16 lactobacilli and fluorescent protein combinations, as follows: *L. plantarum*, IRFP; *L. crispatus*, GFP; *L. gasseri*, mCherry; and *L. jensenii*, mTagBFP2 (Figure 4). The fluorescence of the individual strains and their mixtures in the different proportions was measured, and the nontransformed lactobacilli were used as controls (Figure 5). Again, the individual fluorescent species were clearly distinguished by significantly higher fluorescence when using the settings corresponding to the fluorescent protein they expressed (i.e., the relevant excitation and emission wavelengths), and the fluorescence signals correlated with the contents of the individual strain in the mixtures (Figure 5). When using the settings for IRFP and mCherry (i.e., for determination of *L. plantarum* and *L. gasseri*, respectively), no fluorescence was observed for the nontransformed bacteria (i.e., no autofluorescence) or for the other fluorescent lactobacilli. However, stronger autofluorescence of the nontransformed lactobacilli was seen for the measures with the GFP settings (i.e., for *L. crispatus*), with the strongest seen for the measures with the mTagBFP2 settings (i.e., for *L. jensenii*). The autofluorescence was also species specific, whereby *L. plantarum* showed the lowest autofluorescence using the GFP and mTagBFP2 settings. Considerable overlap was observed between *L. crispatus* that expressed GFP and *L. jensenii* that expressed mTagBFP2, where individual bacteria were detected in both of the fluorescence channels.

**Figure 6:**
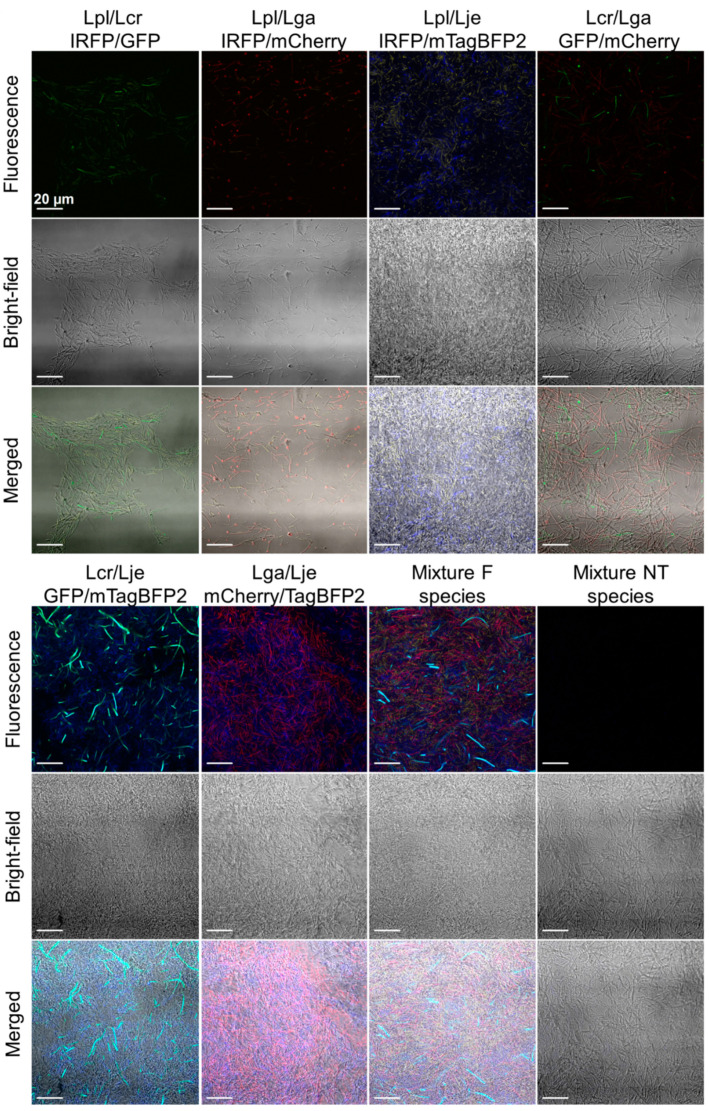
Representative confocal microscopy images of the mixtures of the lactobacilli expressing the different fluorescent proteins (F). Lpl—*L. plantarum*; Lga—*L. gasseri*; Lcr—*L. crispatus*; Lje—*L. jensenii*; NT—nontransformed species. Fluorescence images were obtained by using settings for denoted fluorescent proteins and merging thus obtained images, whereby settings for all four fluorescent proteins were used for the mixture.

However, with confocal microscopy, the majority of the lactobacilli that expressed the different fluorescent proteins were distinguished on the basis of their fluorescence when in mixtures of equal ratios (Figure 6).

### 2.5. Incorporation of Fluorescent Lactobacilli into PEO Electrospun Nanofibers

**Figure 7:**
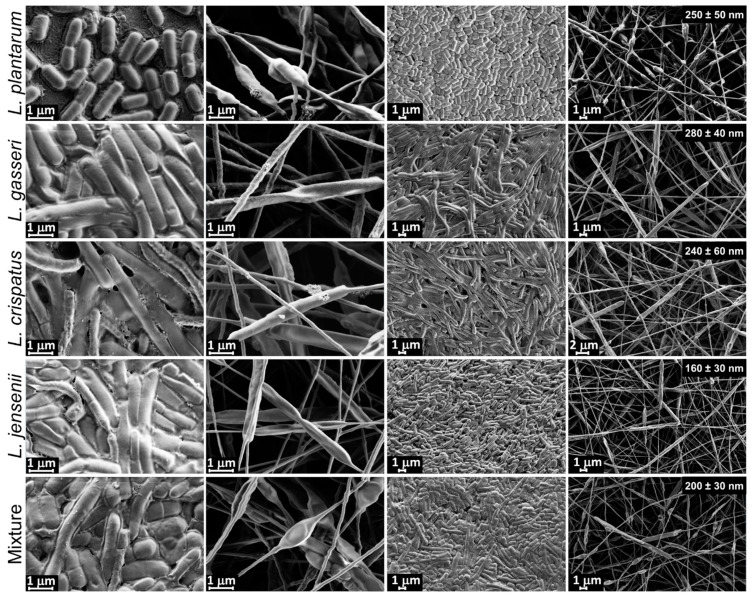
Scanning electron microscopy images of individual lactobacilli and as mixtures under high and low magnification. Columns 1 and 3, air-dried bacteria from water suspensions; Columns 2 and 4, bacteria incorporated into nanofibers. Average nanofiber diameters are specified in the last column.

Fluorescent species of vaginal lactobacilli were successfully incorporated into PEO nanofibers as a potential vaginal delivery system, both individually and as mixtures, including the smallest species (*L. plantarum*: length, 1.28 ± 0.32 µm; width, 0.52 ± 0.04 µm) and the largest species (*L. crispatus*: length, 7.73 ± 1.89 µm; width, 0.81 ± 1.31 µm). The mean diameter of the PEO nanofibers without the bacteria was 170 ±40 nm, and the bacteria incorporation was seen as characteristic thickenings along the nanofibers as reported previously [27,33]. The incorporation of *L. plantarum*, *L. gasseri*, and *L. crispatus* resulted in the increase of the mean nanofiber diameter by app. 100 nm, while the incorporation of *L. jensenii* caused no increase (Figure 7). This may be due to the bacterial release of molecules, such as exopolysaccharides or ions, that can influence the conductivity or viscosity of the polymer suspension.

**Figure 8:**
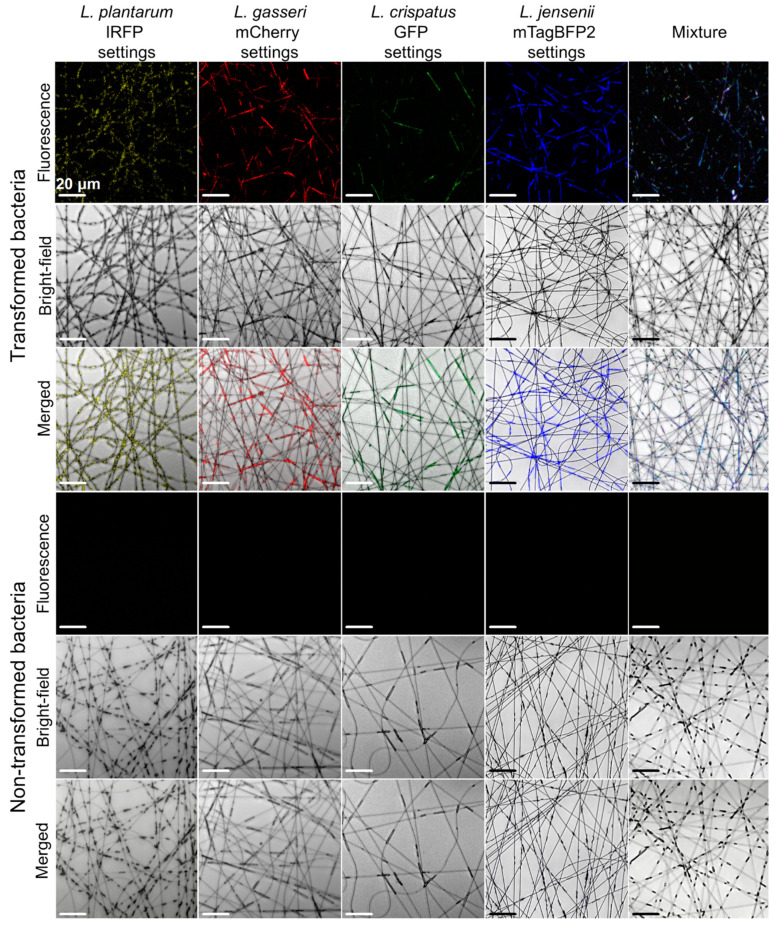
Representative confocal microscopy images of the fluorescent lactobacilli and the nontransformed lactobacilli when incorporated in PEO electrospun nanofibers individually or as mixture of all 4 fluorescent lactobacilli.

Effective incorporation of these bacteria into the nanofibers was also confirmed using confocal microscopy (Figure 8). All of the recombinant species retained their fluorescence following their incorporation in the nanofibers, while no fluorescence was observed for the nontransformed lactobacilli, which were incorporated as the controls. The different lactobacilli could be distinguished in the mixtures on the basis of their fluorescence, although some overlap was observed for *L. crispatus* expressing GFP and *L. jensenii* expressing mTagBFP2. This was similar to the data obtained for the bacterial suspensions (Figure 6).

### 2.6. Release of Lactobacilli from Nanofibers

**Figure 9:**
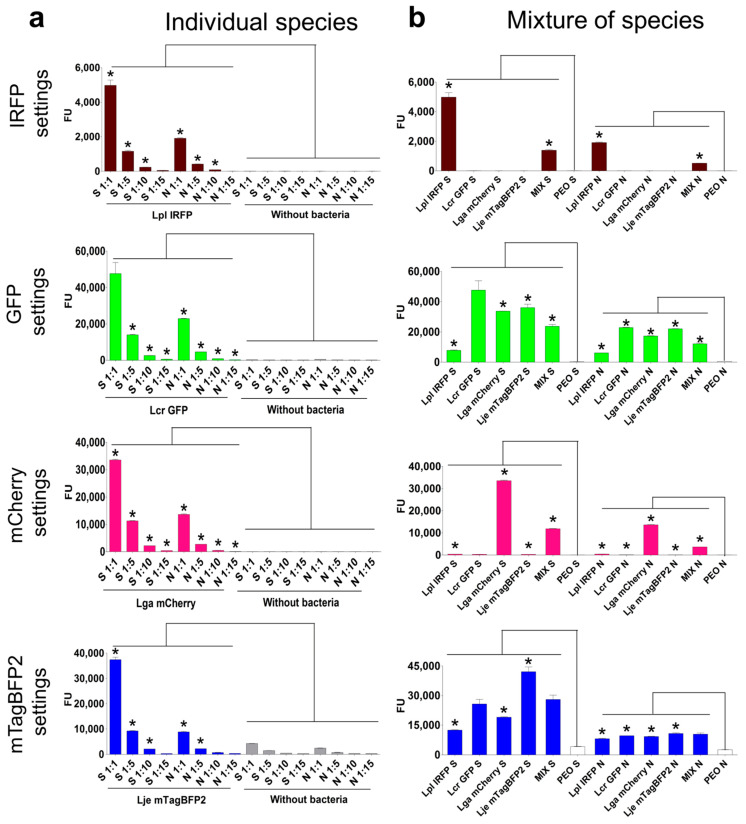
Fluorescence intensities of individual fluorescent *Lactobacillus* species (**a**) and for their mixtures containing all four of the *Lactobacillus* species (**b**), both as dispersed in 4% (*w*/*v*) PEO (S, suspension) prior to incorporation and after their release from the nanofibers (N). Ratios indicate different dilution factors (ratios of the aliquot volume to the final volume). * *p* < 0.05 (Student’s *t* tests), comparison of suspensions or nanofibers containing fluorescent strain to its counterpart without bacteria (**a**), or comparison of suspensions and nanofibers containing fluorescent strains or their mixtures to corresponding PEO control (**b**). PEO—polyethylene oxide.

The lactobacilli from the nanofibers retained their fluorescence after the dissolution of nanofibers. The lower intensity of the fluorescence observed with the dissolved nanofibers was in line with the lower concentration of the lactobacilli in the dissolved nanofibers in comparison to the original dispersions. Namely, the concentration of bacteria per PEO mass was estimated to be on average 3.6-fold higher in suspension than in nanofibers, preventing direct comparison of absolute fluorescence values. Nevertheless, similar to the data above, the fluorescence intensities correlated with the bacterial concentrations in the dissolved nanofibers, as well as in the control 4% (*w*/*v*) PEO bacterial dispersions (Figure 9a). Here, a 4% (*w*/*v*) PEO solution and the dissolved PEO nanofibers without bacteria were used as the negative controls with significantly lower fluorescence. With the exception of *L. jensenii*, the bacteria-containing polymer dispersion and dispersion from nanofibers showed no autofluorescence, regardless of their concentration.

As well as the individual species (Figure 9a), mixtures of all four of these fluorescent lactobacilli were incorporated into PEO nanofibers, with the fluorescence evaluated following the dissolution (Figure 9b). The IRFP-expressing and mCherry-expressing bacteria produced no fluorescence when the settings for the other fluorescent proteins were used. This was not the case for the GFP-expressing and mTagBFP2-expressing bacteria, for which significant fluorescence was observed also when using the settings for the other fluorescent proteins. Additionally, fluorescence overlap was seen, in terms of the fluorescence detected for mCherry-expressing and GFP-expressing bacteria when using the GFP and mTagBFP2 settings, respectively.

## 3. Discussion

To establish methods for imaging of the vaginal lactobacilli, three vaginal *Lactobacillus* species, *L. gasseri* ATCC 33323, *L. crispatus* ATCC 33820, and *L. jensenii* ATCC 25258, and the control *L. plantarum* ATCC 8014 were genetically modified to express fluorescent proteins with different spectral properties: IRFP, GFP, mCherry, and mTagBFP2. This genetic engineering of the vaginal lactobacilli was challenging, particularly for *L. crispatus* [44]. Here, it was performed by electrotransformation using modified and optimized previously published protocols [45,46,47].

Expression of the fluorescent proteins varied between the bacterial species and was highest in the control *L. plantarum*, regardless of the fluorescent protein used. This might be associated with the use of the *ldh* promoter for the control of the transcription of the fluorescent proteins, which originated from *L. plantarum*. The fluorescent intensities of the species were influenced by the growth conditions. According to expectation, higher fluorescence was observed when the lactobacilli were grown with aeration, as the presence of oxygen is crucial for post-translational maturation of the fluorescent proteins, resulting in exo-methylene double bond formation that prevents isomerization [43]. However, these conditions were not favorable for these lactobacilli, which are anaerobes or facultative anaerobes [48,49]. To further improve this approach, anaerobic fluorescent proteins could be considered. Expression of mTagBFP2 affected the growth of *L. plantarum* (not shown), which suggested possible phototoxicity. Nevertheless, all of these species expressed all of these fluorescent proteins, and the fluorescence measurements were proportional to the bacterial concentrations, thus also defining the suitability of this approach for quantifying these bacteria. Very little autofluorescence was seen for the nontransformed bacteria, except when using the mTagBFP2 settings. This contrasted with the expression of mTagBFP2 in *L. rhamnosus*, where autofluorescence was not an issue [42].

The expression of these different fluorescent proteins was also used to distinguish between these different bacterial species in mixtures containing species in equal ratios or in mixtures in which one of the species predominated. This resulted in a very useful tool to gain better insight into the behavior of the lactobacilli in future studies. The fluorescence signals correlated with the contents of the individual strains in the mixtures; however, the overlap between the different fluorescent proteins expressed by the same or different species was also observed. This was particularly evident for mTagBFP2; when using the mTagBFP2 settings, there was considerable fluorescence determined also for the other fluorescent proteins. Further, considerable overlap was observed between *L. crispatus* expressing GFP and *L. jensenii* expressing mTagBFP2, where the individual bacteria were detected in both fluorescence channels. We concluded that fluorescent proteins can be applied to distinguish between vaginal lactobacilli; however, proteins with longer excitation and emission wavelengths (IRFP, mCherry) are more appropriate due to the lower autofluorescence.

Most of the probiotic dosage forms are designed for oral application due to their beneficial effects on the gut. For therapeutic effects in the vagina, intravaginal administration of the probiotics is crucial [50]. To allow intravaginal applications of *Lactobacillus* probiotics, we incorporated these into small diameter fibers, i.e., nanofibers, which were produced using electrospinning [19]. Electrospun nanofibers represent a next-generation delivery system that can be used for biologicals, such as microorganisms, stem cells, proteins, and nucleic acids [23]. The incorporation of bacteria affects the characteristics of nanofibers [33]. Here, a change in diameter was observed, which may be due to the release of bacterial products that can influence the properties of the polymer suspension. Lactobacilli retained their fluorescence after incorporation into nanofibers, as well as after dissolution of the nanofibers, and the fluorescence intensities again correlated with the bacterial concentrations. In bacterial mixtures, the fluorescent proteins with longer excitation and emission wavelengths (i.e., mCherry, IRFP) were clearly distinguished, while for mTagBFP2 and GFP, autofluorescence and spectral overlap interfered with these measurements; this might be resolved with the appropriate compensation. The proposed approach represents a clear advantage over non-specific bacterial staining in pre-formulation and formulation studies of lactobacilli-containing nanofibers.

## 4. Materials and Methods

### 4.1. Bacterial Strains and Culturing

Four different strains from the genus *Lactobacillus* were used in this study: *L. crispatus* ATCC 33820; *L. gasseri* ATCC 33323; *L. jensenii* ATCC 25258; and *L. plantarum* ATCC 8014). *Lactococcus lactis* NZ9000 and *E. coli* DH5α were used as the cloning hosts. Lactobacilli were grown in De Man, Rogosa, and Sharpe (MRS) medium (Merck, Darmstadt, Germany) at 37 °C without and with aeration. *Lc. lactis* NZ9000 was grown in M-17 medium (Merck) supplemented with 0.5% (*v*/*v*) glucose (GM-17) at 30 °C, without aeration. *E. coli* was grown in a lysogeny broth medium at 37 °C, with aeration. All of the strains were kept frozen at −80 °C for long-term storage.

### 4.2. Plasmid Construction

KOD Hot Start DNA polymerase (Merck Millipore, Burlington, MA, USA) was used to fuse the fluorescent protein genes with the *ldh* promoter using overlap-extension PCR [51]. Plasmids pMEC276 [52], pNZ-IRFP713 [39], pCDLbu-1ΔEc-Ptuf34-mCherry [53], and pBAD-mTagBFP2 [54] were used as templates for *ldh*, *IRFP*, *mCherry*, and *mTagBFP2*, respectively. Three individual PCR reactions were prepared using the primers (Integrated DNA Technologies, Leuven, Belgium) specified in Supplementary Material: Table S2. In the first reaction, the promoter and fluorescent protein genes were amplified, and DNA overlaps were introduced (e.g., for *ldh-IRFP* fusion, primer pairs ldh-F/ldh-IRFP-R and IRFP-ldh-F/IRFP-R were used). These two DNA fragments were put in a new PCR mixture without primers to allow direct fusion of the two DNA fragments via complementary overlaps. Finally, in the third PCR reaction, the fused promoter and gene were amplified with a forward primer of the promoter and reverse primer of the fluorescent protein gene (e.g., ldh-F/IRFP-R for *ldh-IRFP*). Gene fusions *ldh-mCherry* and *ldh-mTagBFP2* were prepared in a similar fashion. The DNA products were then inserted into the pJET1.2/blunt vector and transformed into DH5α competent *E. coli* cells. Cloned products were digested using the XbaI/BglII restriction enzymes (Thermo Scientific, Waltham, MA, USA) and ligated into the pNZ8148 [55] plasmid using T4 DNA ligase (Thermo Scientific, Waltham, MA, USA). The three pNZ8148 derivatives thus obtained were: pNZ-ldh-IRFP, pNZ-ldh-mCherry, and pNZ-ldh-mTagBFP2. For the expression of GFP, the plasmid pMEC276 that contained *ldh-GFP* (for clarity, also indicated as pNZ-ldh-GFP) was kindly provided by C. Daniel [52]. The plasmids were transformed into *Lc. lactis* NZ9000 with electroporation using a Gene Pulser II apparatus (Biorad, Hercules, CA, USA), according to the manufacturer instructions (MoBiTec GmbH, Goettingen, Germany). Plasmids were isolated using peqGOLD Plasmid Miniprep Kit I (Peqlabs, Erlangen, Germany) and NucleoSpin Plasmid (Macherey and Nagel, Düren, Germany). Additional treatments with mixtures of lysozyme (50 mg/mL) and mutanolysin were performed when isolating from *Lc. lactis*. Genetic constructs were confirmed by nucleotide sequencing (Eurofins Genomics, Ebersberg, Germany).

### 4.3. Electrotransformation of Lactobacilli

The electrotransformation of lactobacilli was performed with a Gene Pulser II apparatus. Bacterial suspensions (100 µL) were mixed with 5 µL or 10 µL plasmid DNA (200–300 ng/µL) and added to an electroporation cuvette. The species-specific conditions for electroporation and electrocompetent cell preparation are as specified below. After electroporation, 1 mL fresh MRS medium was added to the bacteria, which were then left for 2–3 h to recover, at 30 °C or 37 °C. After the recovery, the bacteria were plated on MRS plates containing 10 µg/mL chloramphenicol (MRSC10). The plates were then placed into anaerobic bags (GasPak^TM^ EZ; Becton Dickinson, Franklin Lakes, NJ, USA) or jars (AnaeroGen^TM^ 2.5l; Thermo Scientific, Waltham, MA, USA) and incubated at 37 °C for 48 h to 72 h.

Electroporation-competent *L. plantarum* was prepared as previously described [45]. Fresh overnight cultures of *L. plantarum* were inoculated in 50 mL MRS medium at 1:50 and grown at 30 °C until an optical density at 600 nm (OD_600_) of 0.4–0.6. The bacteria were then washed twice with 10 mL 10 mM MgCl_2_ and once with 10 mL electroporation buffer (0.5 M sucrose, 10% (*v*/*v*) glycerol). The electroporation parameters for the transformation of *L. plantarum* were: 25 µF, 600 Ω, and 1.8 kV. After electroporation, the bacteria were left to recover at 30 °C for 2 h. A similar protocol was used for *L. jensenii*, with a higher growth and recovery temperature (37 °C) and a higher voltage applied during electroporation (2.4 kV).

To prepare electrocompetent *L. gasseri*, overnight cultures were inoculated in 50 mL MRS medium at 1:50 and grown at 37 °C until an OD_600_ of 0.4. Then, ampicillin (10 µg/mL) was added, and the bacteria were incubated to an OD_600_ of 0.8, followed by three washes with 0.5 M sucrose. *L. gasseri* was electroporated at 25 µF, 400 Ω, and 2.4 kV, and allowed to recover at 37 °C for 3 h, before plating on MRSC10 plates [47].

Overnight cultures of *L. crispatus* were inoculated into 10 mL sterile-filtered MRS medium containing 0.8% (*w*/*v*) glycine, and left for ~10 h at 37 °C until reaching an OD_600_ of 0.5. The bacteria were washed twice with 0.5 M sucrose, incubated on ice with 50 mM EDTA for 5 min, and again washed with 0.5 M sucrose. Electroporation was performed at 25 µF, 600 Ω, and 1.5 kV [46]. Before plating on MRSC10 plates, the bacteria were left to recover at 37 °C for 3 h.

The transformed bacteria were kept frozen at −80 °C in MRS with 20% (*v*/*v*) glycerol for long-term storage.

### 4.4. Culturing of Lactobacilli

Different growth conditions were used for the lactobacilli for the different experiments. Lactobacilli were transferred from frozen stocks to solid MRS media and grown anaerobically at 37 °C for 2–3 days. A single colony was picked and grown in liquid MRS media for 1 day. For measurement of fluorescence, overnight cultures of *Lactobacillus* species transformed with the fluorescent proteins encoded in the pNZ plasmids were inoculated in 5 mL MRSC10 medium and grown at 37 °C without and with aeration, and without and with shaking (180–200 rpm). Biliverdin (15.5 µg/mL; Sigma Aldrich, St. Louis, MO, USA) was added to the medium of species engineered to produce IRFP. The species were grown until late exponential or early stationary phase (OD_600_ 1.5–2.0) when the bacteria were centrifuged at 4400× *g* for 10 min at 4 °C (Centrifuge 5702 R; Eppendorf, St. Louis, MO, USA) and resuspended in phosphate-buffered saline (PBS, pH 7.4) to an OD_600_ of 3.0.

Prior to electrospinning, the engineered bacteria were grown in 400 mL at 37 °C with shaking until reaching OD_600_ of 2.0–3.0. The bacteria were washed twice with water and resuspended in 10 mL water. PEO powder (Mw 900 kDa; Sigma Aldrich, Darmstadt, Germany) was added to the lactobacilli suspensions and stirred at 400 rpm at room temperature until the polymer was completely dissolved to provide the polymer concentration of 4% (*w*/*v*).

### 4.5. Electrospinning of Lactobacilli

Bacterial cells (OD_600_ of 9.0–11.0) were mixed with PEO as described above. The homogenous bacterial-polymer suspensions were filled into a 5 mL syringe that was fixed to an electrospinning machine (Fluidnatek LE100; BioInicia SL, Valencia, Spain). A high voltage of 13 ± 2 kV was applied. The flow rate of the suspension in the syringe was 250–350 µL/h, and the distance between the needle and collector was 15 cm. The electrospinning process was conducted in a climate-controlled environment at 37 °C and 17% relative humidity.

### 4.6. Fluorescence Measurement

The fluorescence of the bacterial suspensions in PBS (200 µL) was measured using a microplate reader (Infinite M1000; Tecan, Männedorf, Switzerland) in 96-well black, flat-bottomed plates. All of the samples were measured in duplicate. Depending on the characteristics of the fluorescent protein, different excitation and emission wavelengths were applied: 402/457 nm for mTagBFP2; 488/509 nm for GFP; 587/610 nm for mCherry; and 690/713 nm for IRFP. The excitation and emission spectra of these fluorescent proteins are provided in Supplementary Material: Figure S1.

To test for correlations between the bacterial concentrations and the fluorescence intensities, serial dilutions of the bacteria were used, corresponding to OD_600_ of 3.0, 2.0, 1.0, 0.5, and 0.25. Nontransformed bacteria were included as the control.

The overlap of the fluorescence signals of the four different fluorescent proteins was assessed by mixing fluorescent strains either in equal ratios (1:1:1:1) or in ratios where individual strain represented 50% of all the bacteria (e.g., 3:1:1:1). These were then compared to the same ratios of the nontransformed bacteria. Individual species that expressed different fluorescent proteins were compared to assign each species with a unique fluorescent protein; selected combinations were *L. plantarum* expressing IRFP, *L. gasseri* expressing mCherry, *L. crispatus* expressing GFP, and *L. jensenii* expressing mTagBFP2, with these compared in a similar fashion. These were also used for confocal microscopy imaging and incorporation into nanofibers.

To measure the fluorescence of the lactobacilli following electrospinning, 10 ± 3 mg PEO nanofibers with the incorporated fluorescent lactobacilli were dissolved in 900 µL PBS and diluted with PBS using the dilution factors (ratios of the aliquot volume to the final volume) of 1:1, 1:5, 1:10, and 1:15. The fluorescence intensities were compared to those of bacterial-polymer suspensions prior to electrospinning, where the concentration of bacteria was estimated to be ~3.6-fold higher. The same dilution factors were applied for the bacterial-polymer suspensions.

### 4.7. Confocal Microscopy

Lactobacilli were grown as described in section Culturing of lactobacilli, resuspended in PBS to an OD_600_ of 3.0, and fixed to a microscope slide with StatSpine Cytofuge 2 (Iris Sample Processing, Westwood, MA, USA) by centrifugation at maximum speed at 4400 rpm for 10 min. The samples were left at room temperature for 1 h to dry and then mounted with a mounting medium (Invitrogen, Waltham, MA, USA) with 4′,6-diamidino-2-phenylindole (DAPI), or IBIDI mounting medium without DAPI. Fluorescent bacteria were visualized with a confocal microscope (LSM-710; Carl Zeiss, Oberkochen, Germany), and images were acquired and processed with the ZEN 2010 B SP1 software (Carl Zeiss, Oberkochen, Germany). The strains were detected with different settings: brightfield, DAPI, Alexa 488, Alexa 543, and Alexa 647, using the 63× immersion oil objective.

For imaging of the nanofibers, a microscope slide was added to the collector of the electrospinning machine, and it was left there for nanofibers to be deposited onto it. A cover slip was added on top and glued with nail polish. Imaging was performed as above, using the 40× immersion oil objective. Nontransformed bacteria were included as the control.

### 4.8. Scanning Electron Microscopy

A scanning electron microscope (Supra 35 VP; Carl Zeiss, Oberkochen, Jena, Germany) was used to visualize the nanofibers with the incorporated bacteria, as well as free bacteria. Individual species and their mixtures were dispersed in water, and 3 µL of the suspension was pipetted onto a metal stub and air-dried. The double-sided conductive tape was used to attach the nanofiber mats to the metal stubs. The scanning electron microscopy was operated at an acceleration voltage of 1 kV, with a secondary electron detector. Bacterial and nanofiber size were analyzed using the ImageJ 1.51j8 software (National Institutes of Health, Bethesda, MD, USA), where the length and width of 30 randomly selected bacteria or nanofibers (regions without bacteria) were measured.

### 4.9. Statistical Analyses

Statistical analyses were performed using the GraphPad Prism 7.0 software, San Diego, CA, USA. Student’s *t* tests were used to define the significances of the differences between the fluorescent bacteria and their respective controls. Calculation of slopes of the regression lines and their comparison was also performed with GraphPad Prism 7.0. All of the data are presented as means ± standard deviation (SD).

## 5. Conclusions

In this paper, we pursued two major goals for wider vaginal probiotics use, namely fluorescent labeling to allow for future distribution studies and designing of an appropriate delivery system. Three of the most important vaginal *Lactobacillus* species, *L. gasseri*, *L. crispatus*, and *L. jensenii*, and the control *L. plantarum* were engineered to produce compatible fluorescent proteins with different spectral properties. The fluorescence intensities were mostly dependent on lactobacilli species and growth conditions. The aeration during culturing promoted the expression of fluorescent proteins compared to samples grown without aeration. The four species were successfully incorporated into nanofibers by electrospinning, which indicated that this technique is appropriate for designing solid nanofiber-based vaginal delivery systems for probiotics. The lactobacilli retained their fluorescence after incorporation into these PEO nanofibers and after their release from them. This research presents a cutting-edge technology to accurately track, by fluorescence imaging, the release of lactobacilli from nanofibers and interactions with the indigenous vaginal microbiota in future in vitro and in vivo studies.

## Data Availability

The data presented in this study are available in the article or its Supplementary Material.

## Supplementary Materials

The following are available online at https://www.mdpi.com/article/10.3390/ijms222413631/s1.
