# Supplementary material for: Engineering of Vaginal Lactobacilli to Express Fluorescent Proteins Enables the Analysis of Their Mixture in Nanofibers
Source: Int J Mol Sci. 2021 Dec 20;22(24):13631. doi: 10.3390/ijms222413631 (PMC8708671; doi:10.3390/ijms222413631)
Supplement: Supplementary file 1 [file ijms-22-13631-s001.zip › ijms-1513001-supplementary.pdf]

Supplementary Material

Engineering of vaginal lactobacilli to express fluorescent proteins enables the analysis of their mixture in nanofibers

Spase Stojanov[1,2], Tina Vida Plavec[1], Julijana Kristl[2], Špela Zupančič[2], Aleš Berlec[1,2,*]

[1] Department of Biotechnology, Jožef Stefan Institute, Ljubljana, Slovenia

[2] Faculty of Pharmacy, University of Ljubljana, Ljubljana, Slovenia

*Correspondence: ales.berlec@ijs.si

Table S1. Coefficients of determination (R^2) and slopes of the linear regression lines. The differences between slopes were determined to be significant in all 16 graphs.

**Species**	**Plasmid**	**Transformed**				**Nontransformed**	
		**Without shaking**		**With shaking**		**With shaking**	
		**R^2**	**Slope**	**R^2**	**Slope**	**R^2**	**Slope**
L. plantarum	pNZ-*ldh*-IRFP	0.9981	$(3.4 \pm 0.1)\times10^2$	0.9994	$(3.7 \pm 0.0)\times10^3$	0.01649	$(0.6 \pm 1.6)\times10^0$
	pNZ-*ldh*-GFP	0.9869	$(1.1 \pm 0.0)\times10^4$	0.9898	$(1.3 \pm 0.0)\times10^4$	0.3166	$(8.7 \pm 4.5)\times10^0$
	pNZ-*ldh*-mCherry	0.9894	$(1.2 \pm 0.0)\times10^4$	0.9974	$(1.4 \pm 0.0)\times10^4$	0.01993	$(-1.2 \pm 3.1)\times10^0$
	pNZ-*ldh*-mTagBFP2	0.9564	$(7.9 \pm 0.6)\times10^3$	0.9984	$(1.3 \pm 0.0)\times10^4$	0.9964	$(2.9 \pm 0.1)\times10^3$
L. gasseri	pNZ-ldh-IRFP	0.9810	$(8.4 \pm 0.4)\times10^1$	0.9985	$(2.9 \pm 0.0)\times10^2$	0.0362	$(-0.5 \pm 1.0)\times10^0$
	pNZ-*ldh*-GFP	0.9929	$(7.4 \pm 0.2)\times10^3$	0.9928	$(1.4 \pm 0.0)\times10^4$	0.9961	$(5.1 \pm 0.1)\times10^1$
	pNZ-*ldh*-mCherry	0.9798	$(2.1 \pm 0.1)\times10^3$	0.9972	$(5.7 \pm 0.1)\times10^3$	0.6565	$(6.3 \pm 1.6)\times10^0$

	pNZ-*ldh*-mTagBFP2	0.9908	$(7.7 \pm 0.2)\times10^{3}$	0.9611	$(1.5 \pm 0.1)\times10^{4}$	0.9996	$(2.1 \pm 0.0)\times10^{3}$
L. crispatus	pNZ-*ldh*-IRFP	0.7996	$(1.4 \pm 0.2)\times10^{1}$	0.9732	$(2.6 \pm 0.1)\times10^{1}$	0.5713	$(3.6 \pm 1.1)\times10^{0}$
	pNZ-*ldh*-GFP	0.9920	$(8.7 \pm 0.2)\times10^{3}$	0.9826	$(1.3 \pm 0.1)\times10^{4}$	0.9935	$(1.8 \pm 0.1)\times10^{3}$
	pNZ-*ldh*-mCherry	0.9638	$(8.0 \pm 0.5)\times10^{1}$	0.9885	$(1.7 \pm 0.1)\times10^{2}$	0.8497	$(8.1 \pm 1.2)\times10^{0}$
	pNZ-*ldh*-mTagBFP2	0.9964	$(8.5 \pm 0.1)\times10^{3}$	0.9984	$(1.3 \pm 0.0)\times10^{4}$	0.9981	$(6.2 \pm 0.9)\times10^{2}$
L. jensenii	pNZ-*ldh*-IRFP	0.9943	$(5.8 \pm 0.2)\times10^{1}$	0.9891	$(6.5 \pm 0.2)\times10^{1}$	0.0038	$(0.1 \pm 0.8)\times10^{0}$
	pNZ-*ldh*-GFP	0.9983	$(7.5 \pm 0.1)\times10^{3}$	0.9983	$(1.4 \pm 0.0)\times10^{4}$	0.9992	$(2.7 \pm 0.0)\times10^{2}$
	pNZ-*ldh*-mCherry	0.9751	$(3.6 \pm 0.2)\times10^{2}$	0.9854	$(6.8 \pm 0.2)\times10^{2}$	0.4361	$(5.4 \pm 2.2)\times10^{0}$
	pNZ-*ldh*-mTagBFP2	0.9927	$(1.2 \pm 0.0)\times10^{4}$	0.9882	$(1.4 \pm 0.1)\times10^{4}$	0.9994	$(5.0 \pm 0.0)\times10^{2}$

Table S2. Primers used in this study.

**Primer name**	**Primer sequence (5'-3')**
ldh-F	AGATCTTCCAACATTATGACG
ldh-IRFP-R	GCTACAGATCCCTCAGCCATAATAAGTCATCCTCTCGTAG
ldh-mCherry-R	TCTTCACCCTTTGATACCATAATAAGTCATCCTCTCGTAG
ldh-mTagBFP2-R	CTCCTTAATCAGCTCGCTCATAATAAGTCATCCTCTCGTAG
IRFP-ldh-F	CTACGAGAGGATGACTTATTATGGCTGAGGGATCTGTAGC
mCherry-ldh-F	CTACGAGAGGATGACTTATTATGGTATCAAAGGGTGAAGAAG
mTagBFP2-ldh-F	CTACGAGAGGATGACTTATTATGAGCGAGCTGATTAAGGAGAAC
IRFP-R	TCTAGATTATTCTTCCATTACACCAATTTGC
mCherry-R	TCTAGATTATCACTGTATAATTCATCC
mTagBFP2-R	TTTATCTAGATTAATTAAGCTTGTGCCCCAGTTTGC

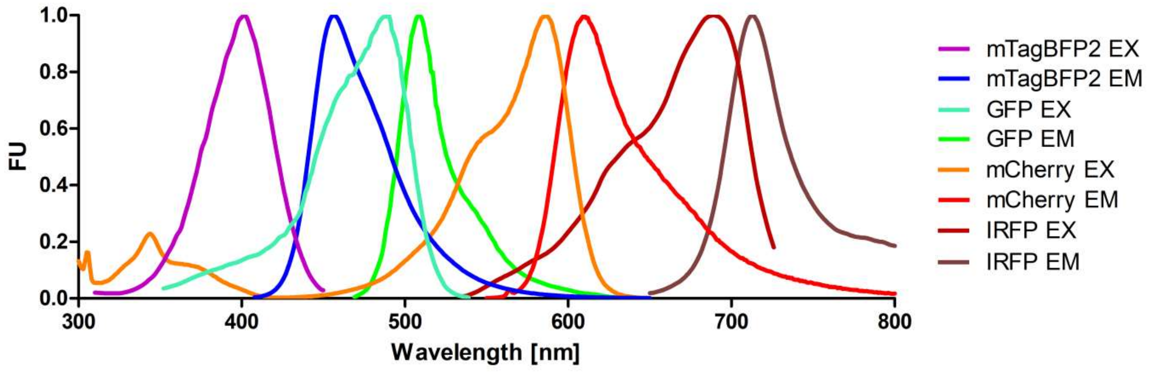

Figure S1. The excitation (EX) and emission (EM) spectra of fluorescent proteins mTagBFP2, GFP, mCherry and IRFP. The data was obtained from FPbase (Lambert, 2019).

Supplementary references:

1. Lambert, T.J. FPbase: a community-editable fluorescent protein database. *Mat Meth* **2019**, 16, 277-278. doi:10.1038/s41592-019-0352-8.
